# Neural Correlates of Facial Expression Recognition in Earthquake Witnesses

**DOI:** 10.3389/fnins.2019.01038

**Published:** 2019-09-27

**Authors:** Francesca Pistoia, Massimiliano Conson, Mario Quarantelli, Luca Panebianco, Antonio Carolei, Giuseppe Curcio, Simona Sacco, Gennaro Saporito, Ernesto Di Cesare, Antonio Barile, Carlo Masciocchi, Alessandra Splendiani

**Affiliations:** ^1^Department of Biotechnological and Applied Clinical Sciences, Neurological Institute, University of L’Aquila, L’Aquila, Italy; ^2^Developmental Neuropsychology Laboratory, Department of Psychology, University of Campania Luigi Vanvitelli, Campania, Italy; ^3^Institute of Biostructure and Bioimaging, National Research Council, Naples, Italy; ^4^Department of Biotechnological and Applied Clinical Sciences, University of L’Aquila, L’Aquila, Italy

**Keywords:** earthquake, emotional, fMRI, visual network, default-mode network

## Abstract

Major adverse events, like an earthquake, trigger different kinds of emotional dysfunctions or psychiatric disorders in the exposed subjects. Recent literature has also shown that exposure to natural disasters can increase threat detection. In particular, we previously found a selective enhancement in the ability to read emotional facial expressions in L’Aquila earthquake witnesses, suggesting hypervigilance to stimuli signaling a threat. In light of previous neuroimaging data showing that trauma exposure is related to derangement of resting-state brain activity, in the present study we investigated the neurofunctional changes related to the recognition of emotional faces in L’Aquila earthquake witnesses. Specifically, we tested the relationships between accuracy in recognizing facial expressions and activity of the visual network (VN) and of the default-mode network (DMN). Resting-state functional connectivity (FC) with the main hub of the VN (primary, ventral, right-dorsal, and left-dorsal visual cortices) and DMN (posterior cingulate/precuneus, medial prefrontal, and right and left inferior parietal cortices) was investigated through a seed-based functional magnetic resonance imaging (fMRI) analysis in both earthquake-exposed subjects and non-exposed persons who did not live in an earthquake-affected area. The results showed that, in earthquake-exposed subjects, there is a significant reduction in the correlation between accuracy in recognizing facial expressions and the FC of the dorsal seed of the VN with the right inferior occipito-temporal cortex and the left lateral temporal cortex, and of two parietal seeds of DMN, i.e., lower parietal and medial prefrontal cortex, with the precuneus bilaterally. These findings suggest that a functional modification of brain systems involved in detecting and interpreting emotional faces may represent the neurophysiological basis of the specific “emotional expertise” observed in the earthquake witnesses.

## Introduction

After a natural disaster, like an earthquake, people usually experience different kinds of emotional dysfunctions or disorders. Among post-earthquake psychiatric complications, the most frequently reported include post-traumatic stress disorder (PTSD), depression, anxiety, obsessive-compulsive disorders, and social phobia (Farooqui et al., 2017; Dube et al., 2018; Geng et al., 2018; Rafiey et al., 2019). The risk of sleep disorders and of prolonged grief symptoms also seems to be increased in earthquake-exposed subjects (Tang et al., 2018; Eisma et al., 2019). On the other hand, experiencing a natural disaster may trigger post-traumatic growth, which refers to positive personality changes following threatening life events and a higher level of functioning (Calhoun and Tedeschi, 2006). Therefore, the spectrum of trauma-related changes may encompass a wide range of manifestations, with subjects who have a poor coping ability being more likely to develop psychiatric disorders and subjects with greater adaptability being able to obtain advantages from adverse events. While the former experience a decreased quality of life after a natural disaster and often need specific psychological or pharmacological support, the latter may thrive (O’Leary and Ickovics, 1995). Independent of developing a clear psychopathological condition, such as PTSD, convergent evidence suggests that trauma exposure increases threat detection (Hayes et al., 2012; Zhang et al., 2014; Bell et al., 2017; Pistoia et al., 2018). In a relevant study on earthquake-exposed persons, Bell et al. (2017) demonstrated that both individuals who develop PTSD and individuals without PTSD are significantly more accurate than non-exposed controls in recognizing emotional facial expressions. The authors interpreted their results in terms of increased sensitivity to threat due to the prolonged exposure to aftershocks in the earthquake-exposed groups.

In this context, we recently reported on a specific emotional expertise developed by earthquake-exposed subjects without PTSD (Pistoia et al., 2018). In detail, we compared two groups of students, one with a permanent residence in the earthquake epicenter of L’Aquila (central Italy) on April 6, 2009, and one not living in an earthquake-affected area. Participants took part in two behavioral experiments aimed at evaluating their ability to recognize facial expressions and to evaluate emotionally evocative scenes. The results demonstrated that students living in the earthquake-affected areas were significantly more accurate than controls in recognizing facial expressions, whereas the two groups did not differ in the evaluation of emotionally evocative scenes. This enhanced recognition ability was not selective for specific emotions, at variance to what has previously been found in neurological patients (Mather and Carstensen, 2005; Pistoia et al., 2010). However, both positive (happiness, surprise) and negative (disgust, fear, anger, sadness) emotions were involved. These results were interpreted in terms of hypervigilance in respect of threats in earthquake witnesses, since trauma exposure, especially in an environment of ongoing threat, requires individuals to maintain their safety by systematically paying attention to potential signs of approaching threat, such as emotional facial expressions (Bell et al., 2017). This interpretation is even more convincing if we consider that the earthquake-exposed students living in L’Aquila experienced a long-lasting exposure to earthquakes with massive psychological distress, as the main event in 2009 was followed by hundreds of thousands of aftershocks in the months afterward and by additional earthquakes in 2016 and 2017.

Recently, Kleshchova et al. (2019) tested the hypothesis that since chronic hypervigilance is a persistent rather than a reactive state, brain correlates can be directly observable under resting-state conditions without the need for exposure to affectively charged stimuli. Results actually showed that, compared to no-trauma controls, trauma-exposed women showed greater connectivity between the amygdala and the cingulate cortex not only during affective processing but also at rest. Long et al. (2014) also demonstrated that testing functional brain changes using resting-state functional magnetic resonance imaging (rs-fMRI) is a useful approach in classifying people who have survived an earthquake who develop psychological responses to trauma exposure. Furthermore, although earthquake witnesses, especially those without PTSD, may not have structural brain changes shortly after the trauma, functional brain changes have been demonstrated as occurring in as little as 1 month after an earthquake (Lui et al., 2009). Against this background, in the present study, we used rs-fMRI to investigate the neurofunctional basis of enhanced recognition of facial expressions in earthquake witnesses.

Data from different neurofunctional approaches demonstrate that the processing of facial expressions crucially involves central nodes of the visual network (VN), like the inferior occipito-temporal cortex, the fusiform and the lingual gyrus, and the lateral temporal cortex (Haxby et al., 2000; Gorno-Tempini et al., 2001; Ganel et al., 2005; Said et al., 2011). Several studies also support the role of the default-mode network (DMN) in interpreting emotional faces, with structures including the medial prefrontal and parietal cortices (Phillips et al., 1998; Harmer et al., 2001; Mattavelli et al., 2011, 2016); the DMN is especially relevant in conditions where explicit expression processing is required (Mattavelli et al., 2016; Zhao et al., 2017). Importantly, the involvement of the VN and the DMN has been demonstrated in trauma-exposed persons during processing of arousing stimuli (Fani et al., 2012; Todd et al., 2015).

By capitalizing on the above evidence, here we used a subsample of Pistoia et al.’s (2018) group of L’Aquila earthquake witnesses to test resting-state functional connectivity (FC) within the major hubs of the VN and the DMN and, crucially, to test FC as it relates to behavioral performance in facial expression recognition task (Pistoia et al., 2018). We hypothesized that enhanced recognition of facial expressions in earthquake-exposed persons could imply an altered pattern of FC between the seeds of the VN and DMN and brain regions related to detection and interpretation of emotional facial expressions, such as the inferior occipito-temporal cortex, the lateral temporal cortex, and the medial parietal cortex.

## Materials and Methods

### Participants

The original sample of the main neuropsychological study by Pistoia et al. (2018) included 107 students, 48 belonging to the experimental earthquake-exposed group with a permanent residence in L’Aquila at the time of the 2009 earthquake (20 males and 28 females, mean age = 22.6, *SD* = 2.3 years) and 59 belonging to the control group not living in an earthquake-affected area (30 males and 29 females, mean age = 23.1, *SD* = 1.6 years). The original inclusion criteria were: (i) no history of previous or coexistent neurological or psychiatric diseases including PTSD, as revealed by a psychiatric examination; (ii) no assumption of drugs or substances acting on the central nervous system; and (iii) signed informed consent to participate in the study.

Here, a subsample was selected from both the earthquake-exposed and the non-exposed group to perform the rs-fMRI study. MRI assessment was restricted to a subsample of subjects because neuroimaging assessment requires a longer time to organize and complete, and not all the subjects originally included were available at the time of the neurofunctional assessment. Following selection, 41 (38%) subjects were included, 18 students belonging to the earthquake-exposed group (8 males and 10 females, mean age = 24.5, *SD* = 1.8 years) and 23 students belonging to the non-exposed control group (14 males and 9 females, mean age = 23.7, *SD* = 2.0 years); non-parametric between-group comparisons showed that the two groups did not differ with respect to both sex and age (both *p* > 0.05).

The research protocol was approved by the Internal Review Board of the University of L’Aquila (January 2017). The study was conducted in accordance with the ethical standards of the Helsinki Declaration and signed informed consent was obtained from all the participants.

### Methods

#### Self-Report Measures

All participants were assessed by means of a series of formalized self-report measures. The Beck Depression Inventory (BDI; Beck, 1967) is one of the most widely used self-report measures for the assessment of depression severity. The score can range from 0 to 63, with higher scores indicating an increasing level of depressive symptoms. The score is usually taken as a dependent variable. The State–Trait Anxiety Inventory (STAI; Spielberger et al., 1983; Pedrabissi and Santiniello, 1989) is a commonly used measure of trait and state anxiety: here, only the 20 items for the assessment of trait anxiety were used. The score can range from 20 to 60; a high score reflects a high level of anxiety. This score was used as a dependent variable. The Insomnia Severity Index (ISI; Bastien et al., 2001; Castronovo et al., 2016) is a self-report questionnaire evaluating different dimensions of insomnia (sleep onset, sleep maintenance and early morning awakening problems, sleep dissatisfaction, interference with daytime functioning, noticeability of sleep problems by others, and distress caused by the sleep difficulties). The score ranges from 0 to 28, with higher scores indicating higher severity of insomnia symptoms; the score was taken into consideration as a dependent variable. The Tolerance of Uncertainty Scale Short Form (IUS-12; Freeston et al., 1994) measures responses to uncertainty, ambiguous situations, and the future. It provides a measure of both prospective anxiety and inhibitory anxiety, as well as a total measure of uncertainty (by summing the scores to all the 12 items). We considered the total score as a dependent variable. The Uncertainty Response Scale (URS; Greco and Roger, 2001) is a scale for the evaluation of styles of coping with uncertainty and can provide a measure of three subscales (emotional uncertainty, desire for control, and cognitive uncertainty). We considered the three subscale scores and the total score as dependent variables. The Anxiety Sensitivity Index 3 (ASI-3; Taylor et al., 2007; Petrocchi et al., 2015) measures vulnerability to anxiety. Higher scores reflect higher levels of anxiety. We considered the physical concerns, social concerns, and cognitive concerns subscales as well as the total score (sum of all the three subscales) as dependent variables. Finally, the Eysenck Personality Questionnaire-Revised Short Form (EPQ-RS; Eysenck et al., 1985; Picconi et al., 2018) was used to assess the personality characteristics of participants. In particular, here we used the scores for neuroticism, extraversion/introversion, and psychoticism scales as dependent variables.

#### Recognition of Facial Expressions Task

In Pistoia et al.’s (2018) study, participants also took part in behavioral experiments aimed at evaluating their ability to recognize facial expressions (using the Ekman and Friesen Pictures of Facial Affect) and to evaluate emotionally evocative scenes (using the International Affective Picture System). In the present study, we specifically focused on the participants’ accuracy in recognizing emotional facial expression, that is the ability to correctly identify actors from the Ekman and Friesen (1976) set of Pictures of Facial Affect (Ekman, 1993) displaying the six basic emotions: happiness, sadness, anger, fear, disgust, and surprise [see Pistoia et al. (2018) for a detailed description of the experimental procedure].

### rs-fMRI

#### Data Acquisition

Magnetic resonance imaging studies were carried out at three Tesla (Discovery MR, General Electric Medical Systems, Erlangen, Germany), using a 32-channel head coil.

Structural T1w volumes were acquired using a three-dimensional magnetization-prepared fast spoiled gradient echo sequence (144 sagittal partitions; TR 6.6 ms; TE 2.3 ms; TI 1100 ms; flip angle 7°; voxel size 1 × 1 × 1 mm^3^).

Resting-state functional magnetic resonance imaging data were acquired using an EPI sequence (50 contiguous axial slices, TR = 3000 ms, TE = 33 ms, FOV = 240 mm, 64 × 64 matrix, slice thickness 3.6 mm, 120 time-points).

In addition, turbo-spin-echo FLAIR axial images were acquired (144 sagittal partitions; TR 8000 ms; TE 119 ms; TI 2032 ms; flip angle 90°; voxel size 1 × 1 × 1 mm^3^) to help rule out the presence of chronic cerebrovascular disease or other CNS pathologies.

During the MRI study, the subjects were lying in a supine position with the head lightly fixed by straps and foam pads to minimize head movement. They were asked to relax with their eyes closed but not to fall asleep during the examination.

#### Data Processing

Magnetic resonance imaging data were pre-processed and analyzed using a toolbox for FC data analysis (CONN – FC toolbox v18b, Gabrieli Lab., McGovern Institute for Brain Research, Massachusetts Institute of Technology^1^; Whitfield-Gabrieli and Nieto-Castanon, 2012) running in Matlab (MathWorks Inc.). CONN is a toolbox for fMRI analysis based on libraries from the Statistical Parametric Mapping package (SPM12, the Wellcome Department of Neurology, London, United Kingdom). Brain tissue probability maps were used to derive the white matter and Cerebro Spinal Fluid (CSF) mean signal time-courses for fMRI pre-processing (see the section “Data Processing”) and to restrict the definition of seeds to each subject’s GM voxels. To this end, for each subject, GM, WM, and CSF probability maps, normalized to the Montreal Neurological Institute (MNI) space, were obtained using the unified segmentation (Ashburner and Friston, 2005), implemented in SPM12. For all the segmentation preprocessing steps, the default SPM12 parameters were used. rs-fMRI pre-processing steps included the following: exclusion of the first five time-points to avoid the effects of the possible instability of the initial MRI signal; correction for differences in acquisition time across slices; motion correction by rigid-body co-registration of all the time-points to the first EPI volume (Friston et al., 1996); and band-pass filtering (0.008–0.09 Hz, to remove low-frequency signal drifts related to scanner instability and high frequency noise). rs-fMRI data were then normalized to the standard MNI space by first coregistering to the corresponding T1-weighted volumes (to avoid misregistration related to inter-sequence movements) and then applying the normalization parameters calculated for the T1 volumes in the segmentation step to the co-registered EPI volumes.

Normalized EPI volumes were then resampled to a voxel size of 3 × 3 × 3 mm^3^. A rigorous removal of signal contributions from head movements and from physiological variations unrelated to neuronal activity was implemented by regressing out the mean white matter and cerebro-spinal fluid signals (Whitfield-Gabrieli and Nieto-Castanon, 2012), along with six framewise motion parameters derived from the motion correction routine (i.e., rotations and shifts along the three orthogonal main axes).

In addition, a “scrubbing” procedure (Power et al., 2012) was applied, consisting of the introduction of dummy regressors to censor the effect of frames with excessive movements and/or signal changes. Accordingly, for each EPI sequence, volumes were identified that had, compared to the previous one, a mean signal difference exceeding three *Z*-values, and/or a mean framewise displacement exceeding 0.5 mm.

On average, 10.1 ± 9.7 (mean ± SD) time-points were removed due to the scrubbing procedure (10.3 ± 10.2 in the controls, 9.7 ± 9.2 in the exposed; *p* = not significant). Analogously, neither mean framewise displacement (0.15 ± 0.11 mm vs. 14 ± 0.07 mm) nor mean global signal change *Z*-values (1.1 ± 0.5 vs. 9 ± 0.2) were significantly different between the two groups.

Functional magnetic resonance imaging volumes were finally smoothed with an isotropic Gaussian filter of 8 mm (FWHM). Voxel-wise maps of FC were then generated by calculating the Fisher-transformed Pearson correlation coefficients between the time course of each voxel and the time course averaged over each of eight seeds, sampling the major hubs of the VN (primary, ventral, right-dorsal, and left-dorsal visual cortices) and of the DMN (posterior cingulate/precuneus, medial prefrontal, right and left inferior parietal cortices). To this end, the seeds provided in CONN were used, which were obtained by independent component analysis of 497 normal subjects from the human connectome project dataset^2^, after masking by the GM map of each patient. The size and the MNI coordinates of the centers of mass of these eight seeds are reported in Table 1.

**TABLE 1:** XYZ coordinates in the MNI space of the center of mass and size of the eight seeds used for the analysis.

**Network**	**Seed**	**Center of mass MNI coordinates (mm)**	**Size (mm^3^)**
Visual	Primary	2, −79, −12	79,224
	Ventral	0, −93, −4	48,712
	Right dorsal	38, −72, 13	33,968
	Left dorsal	−37, −79, 10	24,832
Default-mode	Posterior cingulate/precuneus	1, −61, 38	38,664
	Medial prefrontal	1, 55, −3	10,768
	Right inferior parietal	47, −67, 29	10,608
	Left inferior parietal	−39, −77, 33	8,328

For all the pre-processing steps, an experienced operator, blind to participants’ clinical conditions, visually assessed accuracy of the segmentation and spatial normalization.

#### Statistical Analysis

For each seed, FC maps were then entered in a second-level analysis. To identify differences between the two groups in the strength of the correlation with the VN, or significant interactions between the group and the correlations of the FC of the seeds of both the VN and the DMN with the selected behavioral scores, FC maps were statistically analyzed using a multiple regression analysis within the general linear model framework. Both contrasts (exposed > non-exposed; non-exposed > exposed) were probed when comparing the two subject groups for both the between-group differences and the interaction analyses. Seed-based fMRI analysis was restricted to voxels falling in a GM mask, obtained thresholding at 0.2 the mean of the GM maps obtained in the segmentation step. For all the analyses, age and sex were included as nuisance covariates in the model, along with the mean framewise displacement derived from the motion correction procedure. Results, corrected for family-wise error (FWE) at cluster level, following a cluster-defining threshold of 0.001, were considered significant when surviving an alpha level of 0.05, corrected according to Bonferroni for the number of tests performed (*p* = 0.01 for the comparison between the two groups and 0.006 for the imaging/clinical correlations).

## Results

Behavioral and rs-fMRI raw data are available upon request to the corresponding author.

### Self-Report Measures and Facial Expression Recognition Task

Participants’ scores on the self-report measures strongly overlapped with Pistoia et al.’s (2018) data (Table 2) in revealing higher scores in earthquake witnesses than in controls on several of the selected measures, although the multivariate results did not show significant effects for group [Pillai’s Trace = 0.363; Wilks’ Lambda = 0.637; *F*(14,24) = 0.978; *p* = 0.501, $\eta_{\text{p}}^{2}$ = 0.363] and for sex [Pillai’s Trace = 0.353; Wilks’ Lambda = 0.647; *F*(14,24) = 0.935; *p* = 0.539, $\eta_{\text{p}}^{2}$ = 0.353]. The group by sex interaction was also not significant (*p* > 0.05).

**TABLE 2:** Scores (mean and SD) of the two groups on the self-reported measures and on the facial expression recognition task.

	**Controls**		**Earthquake witnesses**	
		
	**Mean**	***SD***	**Mean**	***SD***
**Self-report measures**				
ISI	4.52	3.1	5.06	3.15
IUS-12	32.52	10.4	44.22	11.7
URS-total score	121.61	12.9	131.06	14.9
URS-emotional uncertainty	27.43	6.5	34.17	8.8
URS-desire for control	45.52	6.2	44.67	8.7
URS-cognitive uncertainty	48.43	8.3	52.5	9.6
STAI-2	38.52	8.2	40.06	6.4
BDI	5.78	4.5	6.89	4.9
ASI-total score	9.35	6.2	16.56	15.9
ASI-physical concerns	2.35	2.3	4.94	6.9
ASI-cognitive concerns	4.13	3.7	5.33	5.8
ASI-social concerns	2.87	3.3	6.28	5.8
EPQ-R-extraversion/introversion	9.22	2.9	7.78	4.1
EPQ-R-neuroticism	4.74	2.8	6.17	3.6
EPQ-R-psychoticism	3.52	1.8	2.5	1.2
**Recognition of facial expressions**				
Disgust	0.76	0.1	0.91	0.1
Happiness	0.98	0.1	1	0.2
Fear	0.64	0.1	0.76	0.1
Anger	0.77	0.2	0.97	0.2
Surprise	0.91	0.2	0.99	0.2
Sadness	0.71	0.1	0.77	0.2

*ISI, Insomnia Severity Index (Bastien et al., 2001; Castronovo et al., 2016); IUS-12, Tolerance of Uncertainty Scale Short Form (Freeston et al., 1994); URS, Uncertainty Response Scale (Greco and Roger, 2001); STAI, State–Trait Anxiety Inventory (Spielberger et al., 1983; Pedrabissi and Santiniello, 1989); BDI, Beck Depression Inventory (Beck, 1967); ASI-3, Anxiety Sensitivity Index (Taylor et al., 2007; Petrocchi et al., 2015); EPQ-R, Eysenck Personality Questionnaire-Revised Short Form (Eysenck et al., 1985; Picconi et al., 2018).*

The performance on the facial expression recognition task largely confirmed our previous data for the whole sample, demonstrating a higher accuracy by earthquake witnesses than non-exposed persons in recognizing all the six emotional categories (happiness, sadness, anger, fear, disgust, and surprise; percentages of correct responses are shown in Table 2). Indeed, the three-way mixed ANOVA on recognition accuracy, with emotion (disgust, happiness, fear, anger, surprise, and sadness) as a within-subject factor, and with group and sex as between-subject factors, showed a significant main effect of emotion [*F*(5,185) = 11.547, *p* = 0.0001, $\eta_{\text{p}}^{2}$ = 0.238], with recognition of fear (0.70) being less accurate than all other emotions (disgust = 0.83; happiness = 0.99; anger = 0.87; surprise = 0.95; and sadness = 0.74). Importantly, results also showed significant main effects of group [*F*(1,37) = 8.844, *p* = 0.005, $\eta_{\text{p}}^{2}$ = 0.193], with overall accuracy being higher in earthquake witnesses (mean = 0.90, *SD* = 0.26) than in controls (mean = 0.79, *SD* = 0.24), and of sex [*F*(1,37) = 4.369, *p* = 0.044, $\eta_{\text{p}}^{2}$ = 0.106], with females (mean = 0.88, *SD* = 0.26) being more accurate than males (mean = 0.81, *SD* = 0.25). No interaction was significant (all *p* > 0.05).

### rs-fMRI Data

All participants were included in the analysis; when asked if they had fallen asleep even briefly, they all confirmed that they had remained awake the whole time.

**FIGURE 1:**
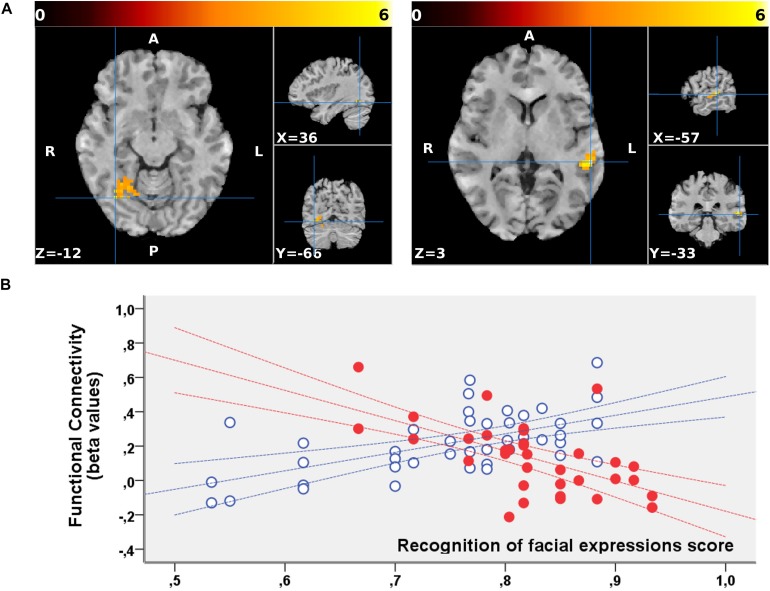
**(A)** Regions showing a significant interaction between the groups (non-exposed > exposed) and correlation of the functional connectivity to the dorsal visual seed of the VN with the behavioral scores for the facial expression recognition task. The two clusters are superimposed to the T1-weighted volume from one of the participants to the study normalized to the MNI space. Color-scale maximum is set to a *T*-value of 6. The three orthogonal planes are centered on the peak values of the two clusters [MNI coordinates, respectively, (57, –33, 3) and (36, –66, –12)]. No significant cluster emerged when probing the inverse (exposed > non-exposed) contrast. **(B)** Corresponding mean FC values are plotted against the scores obtained for the facial expression recognition task for exposed (red, filled marks) and non-exposed (blue, empty marks) subjects. The 95% confidence intervals of the fit are also shown in the corresponding colors. In these regions, the exposed subjects display a significantly inverse correlation between the FC with the left dorsal visual seed and the score, whereas a direct correlation is present in the same regions in non-exposed subjects.

**FIGURE 2:**
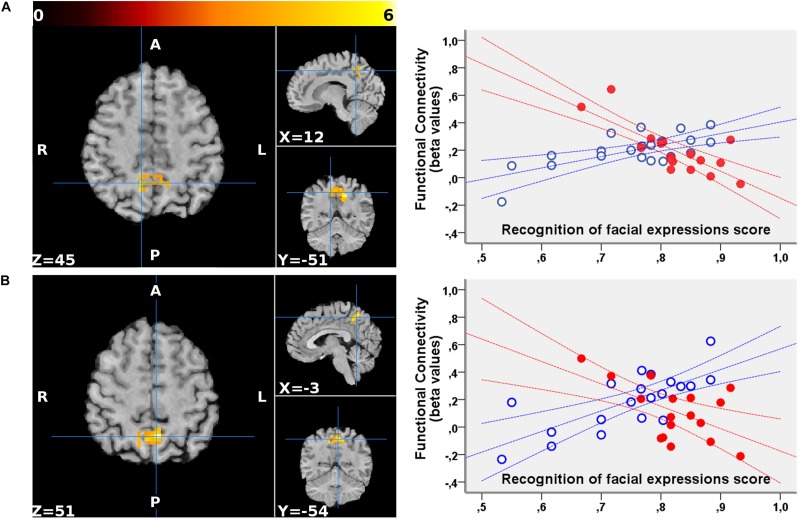
Regions showing a significant interaction between the group and the correlation of the functional connectivity to the medial prefrontal cortex **(A)** and the left lower parietal **(B)** seeds of the DMN with the scores for the facial expression recognition task. For both seeds, the FC with the precuneus (part of the DMN) showed an altered correlation with the scores in the exposed subjects. The clusters are superimposed to the T1-weighted volume from one of the participants to the study normalized to the MNI space. The color-scale maximum is set to a *T*-value of 6. For each cluster, the three orthogonal planes are centered on the peak value MNI coordinates. No significant cluster emerged when probing the inverse (exposed > non-exposed) contrast. On the right, the mean FC values of each cluster are plotted against the scores obtained for the facial expressions recognition task for exposed (red, filled marks) and non-exposed (blue, empty marks) subjects. The 95% confidence intervals of the fit are also shown in the corresponding colors. In the precuneus, the exposed subjects display a significantly inverse correlation between the FC with both these DMN seeds and the score, whereas non-exposed subjects show a direct correlation.

No significant clusters of different FC with any of the tested seeds emerged when comparing the two groups independently of behavioral performance. Differences were found in the between-group correlation of the score for the facial expression recognition task and the FC with the VN and DMN seeds (interaction analysis) (Table 3). For the left dorsal visual seed of the VN, differences emerged in the peripheral ventral occipital cortex on the right (Figure 1A, left) and in the middle temporal gyrus on the left (Figure 1A, right). For seeds of the DMN, differences were found in the precuneus for the medial prefrontal cortex (Figure 2A) and the left lower parietal (Figure 2B). These differences were due to a presence of an inverse correlation between FC and score in these regions in the exposed subjects, as opposed to the direct correlation detectable in the same regions in the non-exposed subjects (Figure 1B and right column of Figure 2).

**TABLE 3:** Clusters of altered correlation of the connectivity with the scores for the facial expression recognition task (Interaction). For each cluster, corresponding *p*-values (corrected at cluster level for family-wise error) and size (in cubic centimeters of gray matter) are reported, along with the local maxima *T*-values and coordinates.

**Network**	**Seed**	***p*-value (FWE)**	**Size (cc)**	***T***	***X***	***Y***	***Z***	**Anatomical labels**
Visual	Left dorsal	<10^–5^	1.4	6.33	–57	–33	3	Left middle temporal gyrus
				4.29	–57	–24	0	Left middle temporal gyrus
				3.82	–51	–18	–6	Left middle temporal gyrus
		<10^–6^	1.8	5.61	36	–66	–12	Right inferior occipital
				5.16	24	–57	–6	Right lingual
				4.39	30	–54	–12	Right fusiform
DMN	Left lower parietal	<10^–13^	4.1	6.68	–3	–54	51	Left precuneus
				6.14	12	–57	45	Right precuneus
				5.72	–6	–48	45	Left precuneus
	Medial prefrontal cortex	<10^–11^	3.7	5.54	12	–51	45	Right precuneus
				5.32	9	–57	39	Right precuneus

*Anatomical labeling is according to Tzourio-Mazoyer et al. (2002).*

## Discussion

The results of the study show significant between-group differences in the correlation of the score for the facial expression recognition task and the FC of the dorsal seed of the VN with the right occipito-temporal cortex and the left middle temporal cortex, and of the two parietal seeds of DMN, i.e., lower parietal and medial prefrontal cortex, with the precuneus bilaterally. These significant between-group differences are consistent with growing data demonstrating chronic and stable changes in functional brain connectivity at rest in persons exposed to trauma (Lui et al., 2009; Long et al., 2014; Chen et al., 2015; Kleshchova et al., 2019). In particular, Kleshchova et al. (2019) suggested that resting neurofunctional changes in trauma-exposed persons are part of an exaggerated neural alerting response to threat that can be observed even in the absence of physical threat, likely due to a chronic trauma-related condition of hypervigilance. It is worth remembering here that our sample of persons exposed to earthquake was actually in a chronic condition since, as noted above, the main L’Aquila earthquake in 2009 was followed by continuous aftershocks in the later months and by additional earthquakes in 2016 and 2017.

The difference we found between exposed and non-exposed participants in the relationship between facial expression recognition and the correlations of FC values in VN and DMN with occipito-temporal, lateral temporal, and medial parietal regions support the idea that the emotional expertise in earthquake witnesses involves neurofunctional changes in networks devoted to the processing of specific signals of potential threats, such as emotional faces. Indeed, although the occipito-temporal cortex represents a central node in the face identity recognition network (Haxby et al., 2000), a recent meta-analysis showed its involvement in the affective representation of a face (Ganel et al., 2005; Said et al., 2011) when both implicit and explicit emotional processing are required (Gorno-Tempini et al., 2001; LeDoux, 2003; Litt et al., 2011; Brooks et al., 2012). Similarly, Mazza et al. (2012) investigated neural response to facial expressions implicitly presented during fMRI in a sample of L’Aquila earthquake witnesses affected by PTSD. Results showed that subliminal presentation of emotional faces (happy and sad) was related to activation of the occipito-temporal cortex, amygdala, and insula. Interestingly, the neurofunctional model of facial processing developed by Haxby et al. (2000) postulates that the core processing system not only involves the ventral occipito-temporal cortex but also the lateral (superior and middle) temporal cortex. Here, we actually found that the behavioral performance of earthquake witnesses was related to altered FC in the VN with the left middle temporal gyrus, consistent with Haxby et al.’s (2000) model, according to which this cortical region would be particularly involved in detecting the changeable aspects of faces, such as emotional expressions.

Facial expressions are actually among the most relevant signals conveying information on what is going on in other persons’ minds (Adolphs, 1999, 2003; LeDoux, 2003; Kanwisher and Yovel, 2006). Many imaging studies have examined the neural basis of understanding others’ minds by different experimental tasks, such as judgments on facial expressions, stories, or moving shapes (Frith and Frith, 2006, 2007). Results always show the activation of a set of regions including medial prefrontal and parietal cortex, and posterior temporo-parietal areas around the temporo-parietal junction (Frith and Frith, 2006, 2007), a network of areas largely overlapping with the DMN (e.g., Mars et al., 2012). In particular, the left parietal and posterior midline nodes of the DMN are involved in processing emotional facial expressions, both in healthy individuals (Sreenivas et al., 2012) and in patients with different psychopathological conditions including social phobia (Gentili et al., 2009) and schizophrenia (Salgado-Pineda et al., 2011). Schilbach et al. (2008) explored the relationship between the neural basis for social cognition and the DMN, and found that the core nodes of the DMN overlap with those involved in social cognition (Vogeley and Fink, 2003; Schilbach et al., 2006). The authors suggested that the resting default state of the human brain is related to the predisposition of humans for social cognition as a default mental state. Consistently, here we demonstrated the involvement of key nodes of the DMN as the lateral parietal cortex, and the medial prefrontal and the parietal cortex. Therefore, we suggest that this default tendency to focus on the other person’s mental state could be enhanced in persons exposed to traumatic experiences, as in the case of earthquake witnesses, in order to search relevant social signals, allowing rapid identification of possible threats in the environment (Zhang et al., 2014; Bell et al., 2017; Pistoia et al., 2018). This result fits with findings from a seminal rs-fMRI study on witnesses of the Wenchun earthquake in China by Lui et al. (2009), who found a reduced temporal synchronization within the DMN in trauma victims, even immediately after trauma exposure. Moreover, a recent study investigating the correspondence between spontaneous neural activity in the DMN and the severity of PTSD symptoms showed that the at-rest activity of the left inferior parietal lobule was positively correlated with symptom severity, thus suggesting that its activity is involved in the cognitive biases observed in persons with PTSD (Disner et al., 2018).

Future studies on earthquake witnesses are warranted to replicate the present results on a large sample; the size of the present group was small, although it was in line with the size of samples recruited in similar studies (e.g., Long et al., 2014; Kleshchova et al., 2019). Further, sensitivity of the study may have been limited by the relatively short scan duration which, however, was within the timeframe that has been shown to be required to stabilize the correlation strengths within and between the major networks (Van Dijk et al., 2010). While we preferred to keep acquisition short to reduce the risk of the subject falling asleep and/or moving, the increased S/N ratio achievable with longer acquisitions may be considered in future studies, to increase sensitivity.

Also, shorter TRs allowed by multiband acquisition (Feinberg and Setsompop, 2013), which was not available on our scanner, may in the future allow further boosting of the S/N ratio, overcoming the limitations derived from the relatively long sampling interval (3 s), which was used here to allow complete brain coverage while keeping a reasonable in-plane resolution with the available hardware.

Notwithstanding these limitations, our findings suggest that emotional expertise in earthquake witnesses goes through a functional modification of brain systems devoted to detection, identification, and interpretation of emotional faces, including the occipito-temporal cortex and the medial parietal cortex. Since we observed a general increase in anxiety and anticipation of threats, as well as emotional uncertainty, such emotional expertise, although first developing as a response of adaptive value, ends up being a maladaptive change to trauma, likely related to anxiety responses (see also Pistoia et al., 2018). It is possible to suggest that this emotional response is even more likely in young persons, in whom traumatic experiences tend to have a great impact on psychological functioning (Wang et al., 2015; Hong and Efferth, 2016). The earthquake witnesses who participated in the present study were young teenagers in 2009. Thus, in future studies, it could be interesting to investigate “emotional expertise” after a natural disaster in persons who were exposed to the traumatic experience at a later stage of their life.

## Data Availability Statement

The datasets generated for this study are available on request to the corresponding author.

## Ethics Statement

The research protocol was approved by the Internal Review Board of the University of L’Aquila (01/2017). The study was conducted in accordance with the ethical standards of the Helsinki Declaration and signed informed consent was obtained from all the participants.

## Author Contributions

All authors equally contributed to the planning, development, and drafting of the manuscript.

## Conflict of Interest

The authors declare that the research was conducted in the absence of any commercial or financial relationships that could be construed as a potential conflict of interest.

## Footnotes

1 http://www.nitrc.org/projects/conn

2 http://www.humanconnectomeproject.org
